# The composition of lung microbiome in lung cancer: a systematic review and meta-analysis

**DOI:** 10.1186/s12866-021-02375-z

**Published:** 2021-11-11

**Authors:** Sadaf Najafi, Fatemeh Abedini, Sadegh Azimzadeh Jamalkandi, Parvin Shariati, Ali Ahmadi, Mohammad Gholami Fesharaki

**Affiliations:** 1 Department of Biostatistics, Faculty of Medical Sciences, Tarbiat Modares University, Tehran, Iran; 2 Department of Bioprocess Engineering, Institute of Industrial and Environmental Biotechnology, National Institute of Genetic Engineering and Biotechnology, Tehran, Iran; 3 Chemical Injuries Research Center, Systems Biology and Poisonings Institute, Baqiyatallah University of Medical Sciences, Tehran, Iran; 4 Molecular Biology Research Center, Systems Biology and Poisonings Institute, Baqiyatallah University of Medical Sciences, Tehran, Iran

**Keywords:** Lung microbiome, Lung cancer, Meta-analysis, 16S rRNA gene, Metagenomics

## Abstract

**Background:**

Although recent studies have indicated that imbalance in the respiratory microbiome composition is linked to several chronic respiratory diseases, the association between the lung microbiome and lung cancer has not been extensively studied. Conflicting reports of individual studies on respiratory microbiome alterations in lung cancer complicate the matter for specifying how the lung microbiome is linked to lung cancer. Consequently, as the first meta-analysis on this topic, we integrate publicly available 16S rRNA gene sequence data on lung tissue samples of lung cancer patients to identify bacterial taxa which differ consistently between case and control groups.

**Results:**

The findings of the current study suggest that the relative abundance of several bacterial taxa including *Actinobacteria* phylum, *Corynebacteriaceae* and *Halomonadaceae* families, and *Corynebacterium*, *Lachnoanaerobaculum*, and *Halomonas* genera is significantly decreased (*p* < 0.05) in lung tumor tissues of lung cancer patients in comparison with tumor-adjacent normal tissues.

**Conclusions:**

Despite the underlying need for scrutinizing the findings further, the present study lays the groundwork for future research and adds to our limited understanding of the key role of the lung microbiome and its complex interaction with lung cancer. More data on demographic factors and tumor tissue types would help establish a greater degree of accuracy in characterizing the lung microbial community which accords with subtypes and stages of the disease and fully capturing the changes of the lung microbiome in lung cancer.

**Supplementary Information:**

The online version contains supplementary material available at 10.1186/s12866-021-02375-z.

## Background

With the advent of culture-independent DNA sequencing technologies and the development of Next Generation Sequencing (NGS) techniques, the previously unknown world of the human microbiome (the entire microorganisms inhabit a specific environment (e.g. the human body) including bacteria, archaea, eurkaryotes, and viruses along with their genomes and surrounding environmental conditions) [1] has been recognized and received considerable attention. Numerous studies have investigated the interplay between the microbiome and the host immune system during health and disease, arriving at a consensus that a dysbiotic microbiome may be correlated with disease onset and progression [2, 3]. Due to the initial assumption considering the lungs as sterile sites, the dynamic changes that may occur in the lower respiratory tract microbiome were previously completely neglected. However, new findings revealing the existence of a low-density yet diverse microbial ecosystem in healthy lungs, have confirmed its critical role during respiratory diseases [4, 5]. It has been demonstrated that an impaired lung microbiome is associated with the development of chronic lung diseases such as chronic obstructive pulmonary disease (COPD) [6–8], cystic fibrosis (CF) [9–11], asthma [12–14], and idiopathic pulmonary fibrosis (IPF) [15–17]. In recent years, the specific impact of the microbiome on lung cancer has gained increasing interest. Lung cancer is one of the most serious lung diseases and common cancer in both men and women. With a high mortality rate (1.6 million annually), lung cancer is the leading cause of cancer death worldwide [18]. The disease is initially asymptomatic and usually diagnosed in advanced stages. Late diagnosis and high mortality rate of lung cancer emphasize the importance of identifying microbial composition and potential signatures according to the stages of the disease. Dozens of studies have relied mostly on 16S rRNA amplicon sequencing approach using different clinical samples including bronchoalveolar lavage (BAL) fluid, sputum, saliva, and lung biopsy to evaluate the contribution of the lung microbiome in relation to lung cancer [19–24]; as the application of shotgun sequencing in the respiratory microbiome is still in its infancy [5]. Although most of the prior investigations have found significant differences in the taxonomic composition of the lung microbiome in the disease state, specifying the microbial profiles and patterns that may contribute to the pathogenesis of the disease is still a major challenge owing to inconsistencies in reported studies. Contradictory results may stem from the lack of a standard pipeline in preprocessing the metagenomic data, differences in study designs, clinical sample types, and computational methods as well as other confounding factors and inter-study batch effects such as experimental procedures, targeted hypervariable regions of 16S rRNA gene for amplification and sequencing platforms [25]. These limitations hinder the generalizability of the results, necessitating the need for meta-analysis studies. To address this, meta-analyses are conducted with the aim of reducing the bias of individual studies, specifically the small sample size, and thus yielding more robust results; as the power of meta-analysis has been demonstrated by recent microbiome meta-analyses which have identified some disease-associated microbial signatures [26–29]. In the present study, as the first meta-analysis of the lung microbiome in lung cancer (LC) patients, we reprocessed and integrated 16S rRNA gene sequence data on lung biopsy specimens with geographically different sample origins across five studies consisting of 356 tumor tissues and 493 tumor-adjacent normal tissues. We have aimed to identify the differences in the microbiome between the two groups and determine the possible associations between the taxonomic composition of the lung microbiome and lung cancer.

## Results

**Fig. 1:**
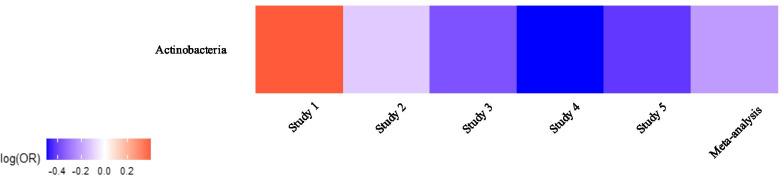
Heat map of changes in the relative abundance of *Actinobacteria* phylum across all studies. Regression coefficients from GAMLSS-BEZI are log (odds ratio) (log(OR)) of changes in the relative abundance of a specific bacterial taxon between the case and control groups and pooled log (OR) estimate is from a random-effects meta-analysis. The case group is considered as the reference group and shown in the heat map. Log(OR) > 0 indicates an increase and log(OR) < 0 indicates a decrease in the relative abundance of *Actinobacteria* in tumor tissues as compared to tumor-adjacent normal tissues

**Fig. 2:**
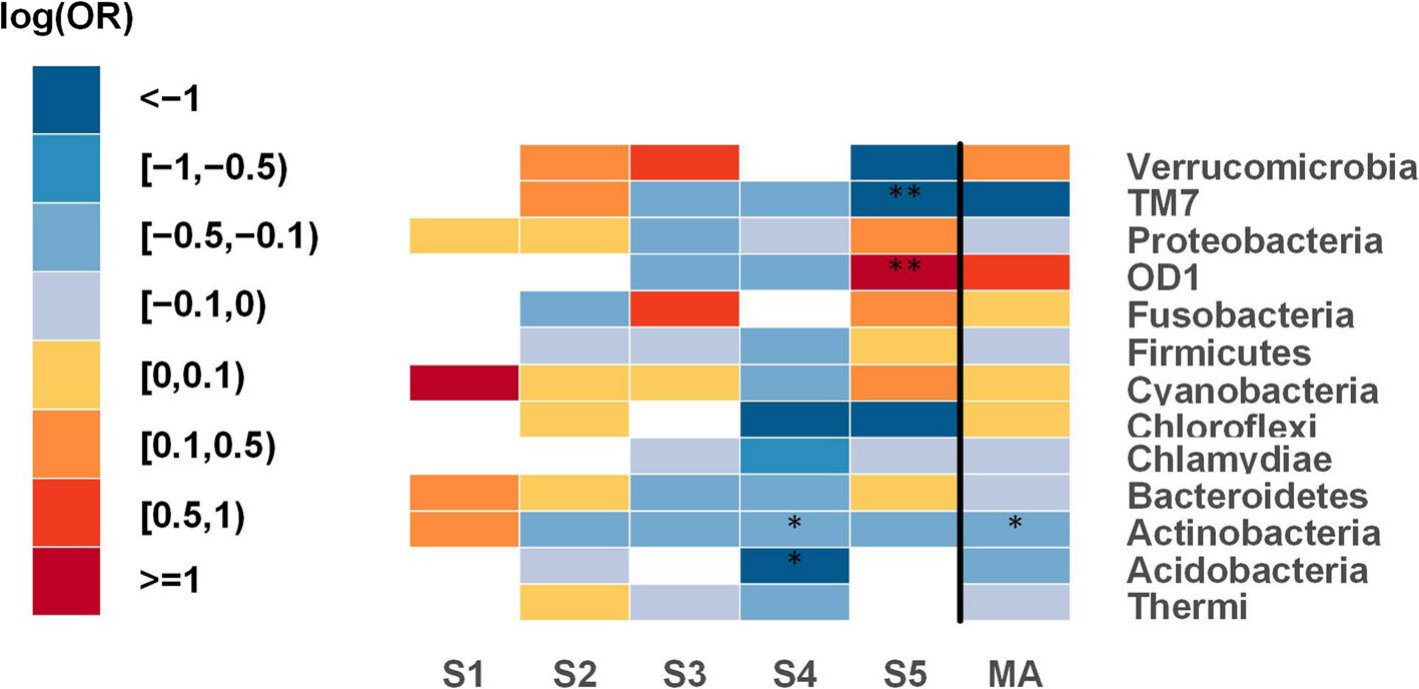
Phylum level meta-analysis between tumor tissues and tumor-adjacent normal tissues; heat map for representing changes of all phyla. Statistically significant differences between the two groups with p-values < 0.05 are denoted with * and those with p-values < 0.0001 are denoted with **. The white parts in the heat map represent the bacterial taxa that are not available in a particular study. S (1-5): Study; MA: Meta-analysis

To investigate the possible changes of the lung microbiome in lung cancer, raw sequence data from a total of five studies were processed into relative abundance data. Samples used in these studies were obtained from patients in different geographic regions, yet subjects were age-homogeneous. To distinguish between cases and controls in terms of taxonomic changes of the lung microbiome, differences between tumor tissues and tumor-adjacent normal tissues were determined using Generalized Additive Models for Location, Scale and Shape (GAMLSS) [30, 31] with a zero-inflated beta distribution (BEZI) within each study. Regression coefficients from the GAMLSS-BEZI model were then retrieved as summary statistics and combined by employing a random-effects meta-analysis model to seek consistent associations between lung cancer and the lung microbiome at various taxonomic levels. At phylum level, there was a significant difference (*p* = 0.016) in *Actinobacteria* between cases and controls (decreased relative abundance in tumor tissues). As can be seen in Fig. 1, although one study with the smallest sample size showed non-significant enrichment in *Actinobacteria* in tumor tissues, four other studies showed decreased relative abundance of *Actinobacteria* in cases vs. controls, with a significant decrease (*p* = 0.039) in study 4. The results obtained from the individual studies and the meta-analysis at the phylum level are presented in Table 1. The changes of all phyla across all studies can be compared in Fig. 2.

**Table 1:** Results of GAMLSS-BEZI and Random Effects Meta-analysis across included studies at the phylum level

Bacterial phylum	Estimate	SE	Lower limit	Upper limit	p-value	Study	Population
	0.40	0.76	-1.10	1.89	0.6130	Study 1	AF & EU
*Actinobacteria*	-0.11	0.07	-0.25	0.03	0.1310	Study 2	IL
	-0.37	0.23	-0.82	0.08	0.1108	Study 3	IT 1
	-0.49	0.23	-0.95	-0.04	0.0386	Study 4	IT 2
	-0.42	0.30	-1.00	0.16	0.1614	Study 5	RU
	-0.21	0.09	-0.39	-0.04	0.0162	Meta_analysis	Pooled

AF & EU: Africa & Europe; IL: Israel; IT: Italy; RU: Russia

**Fig. 3:**
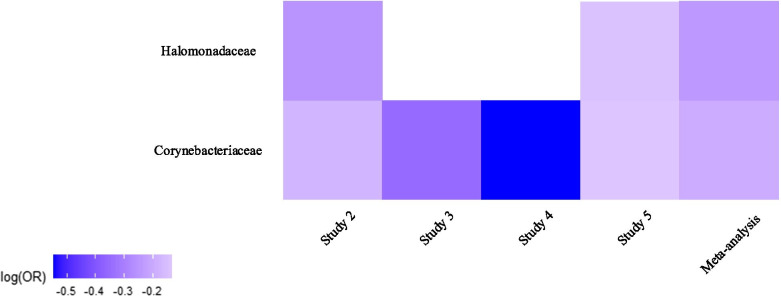
Heat map of changes in the relative abundance of *Corynebacteriaceae* and *Halomonadaceae* families across all studies. Regression coefficients from GAMLSS-BEZI are log (odds ratio) (log(OR)) of changes in the relative abundance of a specific bacterial taxon between the case and control groups and pooled log (OR) estimate is from a random-effects meta-analysis. The case group is considered as the reference group and shown in the heat map. Log(OR) < 0 indicates a decrease in the relative abundance of *Corynebacteriaceae* and *Halomonadaceae* in tumor tissues as compared to tumor-adjacent normal tissues. The white parts in the heat map represent the bacterial taxa that are not available in a particular study

**Fig. 4:**
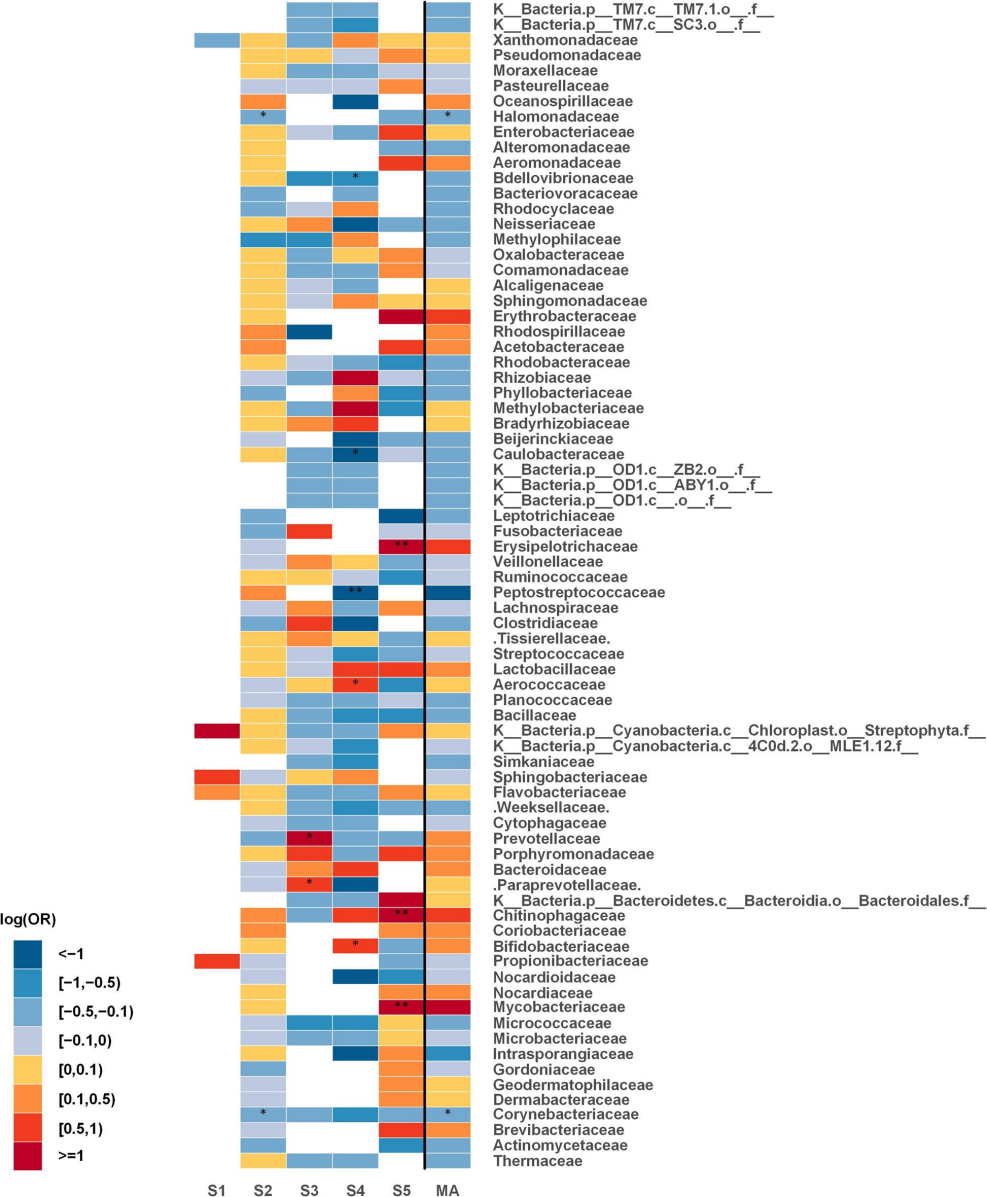
Family level meta-analysis between tumor tissues and tumor-adjacent normal tissues; heat map for representing changes of all families. Statistically significant differences between the two groups with p-values < 0.05 are denoted with * and those with p-values < 0.0001 are denoted with **. The white parts in the heat map represent the bacterial taxa that are not available in a particular study. S (1-5): Study; MA: Meta-analysis

At family level, *Corynebacteriaceae* (*p* = 0.012 across four of the studies) and *Halomonadaceae* (*p* = 0.016 across two of the studies) were significantly decreased in tumor tissues. As shown in Fig. 3, there was a consistent decrease (significantly decreased in study 2) in the relative abundance of both *Corynebacteriaceae* and *Halomonadaceae* in tumor tissues. The results obtained from the individual studies and the meta-analysis at the family level are summarized in Table 2. The changes of all families across all studies can be compared in Fig. 4.

**Table 2:** Results of GAMLSS-BEZI and Random Effects Meta-analysis across included studies at the family level

Bacterial family	Estimate	SE	Lower limit	Upper limit	p-value	Study	Population
						Study 1	AF & EU
*Corynebacteriaceae*	-0.17	0.08	-0.34	-0.01	0.0389	Study 2	IL
	-0.35	0.35	-1.03	0.34	0.3182	Study 3	IT 1
	-0.54	0.39	-1.32	0.23	0.1723	Study 4	IT 2
	-0.13	0.37	-0.86	0.59	0.7210	Study 5	RU
	-0.19	0.08	-0.35	-0.04	0.0124	Meta_analysis	Pooled
						Study 1	AF & EU
*Halomonadaceae*	-0.25	0.11	-0.46	-0.04	0.0180	Study 2	IL
						Study 3	IT 1
						Study 4	IT 2
	-0.14	0.29	-0.72	0.43	0.6279	Study 5	RU
	-0.24	0.10	-0.44	-0.04	0.0165	Meta_analysis	Pooled

AF & EU: Africa & Europe; IL: Israel; IT: Italy; RU: Russia

**Fig. 5:**
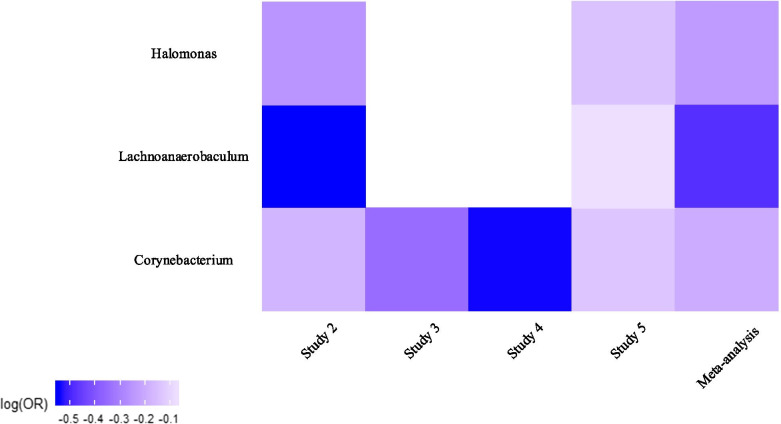
Heat map of changes in the relative abundance of *Corynebacterium*, *Lachnoanaerobaculum*, and *Halomonas* genera across all studies. Regression coefficients from GAMLSS-BEZI are log (odds ratio) (log(OR)) of changes in the relative abundance of a specific bacterial taxon between the case and control groups and pooled log (OR) estimate is from a random-effects meta-analysis. The case group is considered as the reference group and shown in the heat map. Log(OR) < 0 indicates a decrease in the relative abundance of *Corynebacterium*, *Lachnoanaerobaculum*, and *Halomonas* in tumor tissues as compared to tumor-adjacent normal tissues. The white parts in the heat map represent the bacterial taxa that are not available in a particular study

**Fig. 6:**
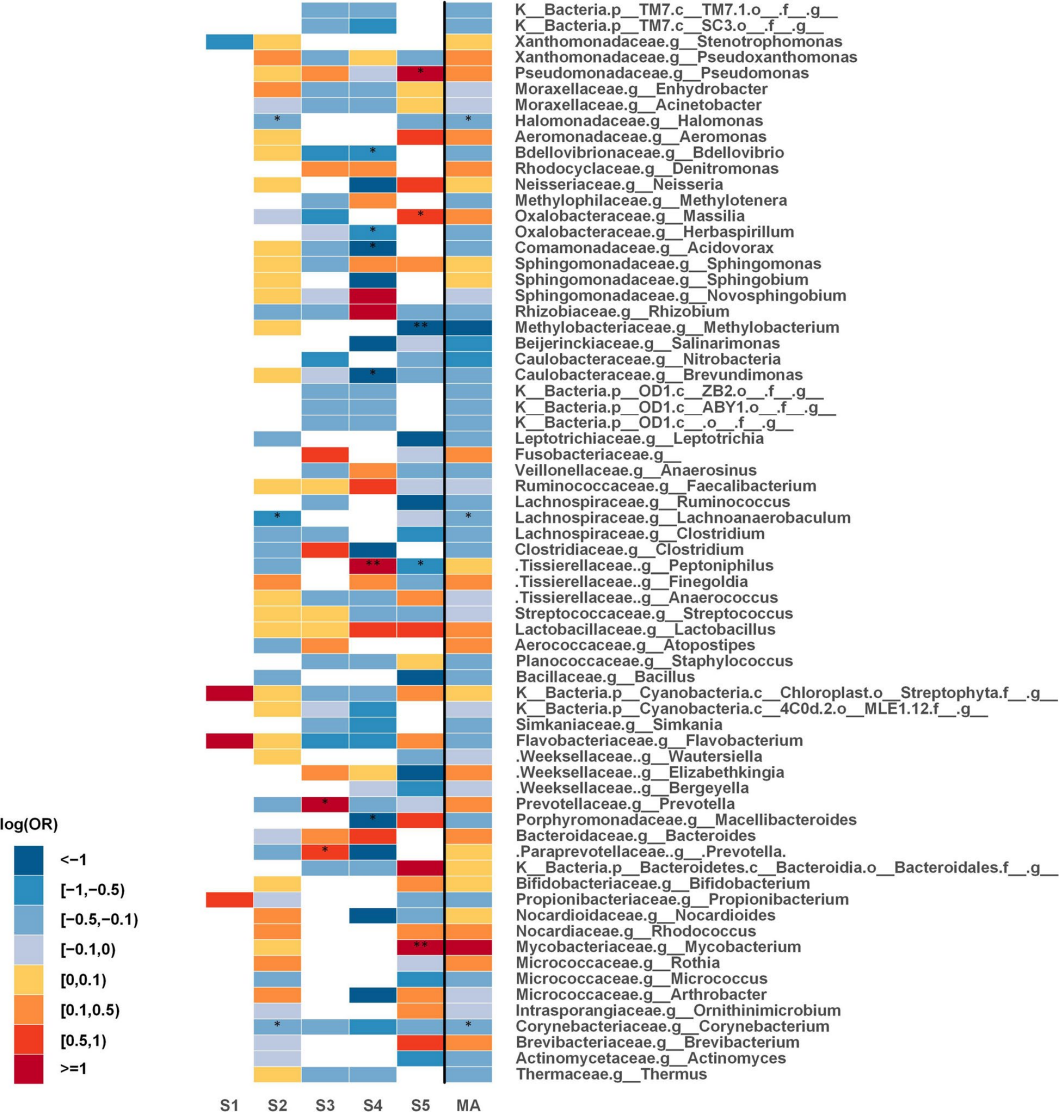
Genus level meta-analysis between tumor tissues and tumor-adjacent normal tissues; heat map for representing changes of all genera. Statistically significant differences between the two groups with p-values < 0.05 are denoted with * and those with p-values < 0.0001 are denoted with **. The white parts in the heat map represent the bacterial taxa that are not available in a particular study. S (1-5): Study; MA: Meta-analysis

At genus level, the relative abundance of three genera was found to be significantly decreased in tumor tissues as compared to controls; *Corynebacterium* (*p* = 0.012 across four of the studies), *Lachnoanaerobaculum* (*p* = 0.015 across two of the studies), and *Halomonas* (*p* = 0.018 across two of the studies). As illustrated in Fig. 5, the direction of changes was consistent across studies. The relative abundance of *Corynebacterium* was consistently decreased in cases relative to controls across the four studies (significantly decreased in study 2). Analogous to that of *Corynebacterium*, the relative abundance of *Lachnoanaerobaculum* and *Halomonas* was also consistently lower in cases across the two studies (studies 2 and 5); with a significant result in study 2. The results obtained from the individual studies and the meta-analysis at the genus level are shown in Table 3. The changes of all genera across all studies can be compared in Fig. 6.

**Table 3:** Results of GAMLSS-BEZI and Random Effects Meta-analysis across included studies at the genus level

Bacterial genus	Estimate	SE	Lower limit	Upper limit	p-value	Study	Population
						Study 1	AF & EU
*Corynebacterium*	-0.17	0.08	-0.34	-0.01	0.0389	Study 2	IL
	-0.35	0.35	-1.03	0.34	0.3182	Study 3	IT 1
	-0.54	0.39	-1.32	0.23	0.1723	Study 4	IT 2
	-0.13	0.37	-0.86	0.59	0.7210	Study 5	RU
	-0.19	0.08	-0.35	-0.04	0.0124	Meta_analysis	Pooled
						Study 1	AF & EU
*Lachnoanaerobaculum*	-0.55	0.21	-0.97	-0.13	0.0106	Study 2	IL
						Study 3	IT 1
						Study 4	IT 2
	-0.07	0.55	-1.15	1.00	0.8926	Study 5	RU
	-0.49	0.20	-0.88	-0.10	0.0147	Meta_analysis	Pooled
						Study 1	AF & EU
*Halomonas*	-0.25	0.11	-0.46	-0.04	0.0199	Study 2	IL
						Study 3	IT 1
						Study 4	IT 2
	-0.14	0.29	-0.72	0.43	0.6279	Study 5	RU
	-0.24	0.10	-0.43	-0.04	0.0181	Meta_analysis	Pooled

AF & EU: Africa & Europe; IL: Israel; IT: Italy; RU: Russia

Taken together, these results suggest that there is an association between lung microbiome dysbiosis and lung cancer.

## Discussion

Although prior studies have shown the major impact of microbiome dysbiosis on respiratory diseases such as COPD, cystic fibrosis, asthma, and idiopathic pulmonary fibrosis, very little was found in the literature on microbiome alterations during lung cancer. Available reports on the microbiome composition specific to lung cancer have been highly inconsistent and therefore the question remains unanswered. In reviewing the literature, no meta-analysis was found on the association between the microbiome and lung cancer, mainly due to the fact that the lung microbiome is considered an emerging research area. Sterility of the lungs is still a matter of controversy. Although microbial culture of healthy individuals’ lower respiratory tract specimens is negative, metagenomics studies have convinced scientists of the existence of genomes of various microorganisms residing in the lungs most of which are not culturable. In this regard, there are some issues that cause studies to obtain different results; including type of clinical sample, different sampling methods, challenges of the process of metagenomic analysis, and the personalized nature of the human microbiome, and therefore identifying and determining the composition and abundance of the lung microbiome in healthy individuals is still a major challenge. Accordingly, as the first meta-analysis aiming to represent taxonomic changes of the lung microbiome in lung tumor tissues, the primary aim of this meta-analysis is to integrate the results of such conflicting studies for better understanding of the alterations in the microbiome content.

Similar to other meta-analyses on microbiome-disease state, we have used cross-sectional studies here. Although the microbiome may be dynamic and change over time as environmental conditions change, the most important part of each individual’s microbiome is stable, namely, the core microbiome (microbial taxa or genes that are stable over time and shared by all or most of the population and are particularly important for the host’s biological function). In fact, the core microbiome is considered as the microbiome’s fingerprint of each individual and most microbiome studies, both individual and meta-analyses, try to identify a pattern of the core microbiome in different people under different conditions. We showed some significant decreases in the relative abundance of several bacterial phyla, families, and genera. Although the phylum *Proteobacteria* and especially the genus *Streptococcus* have been suggested as key bacteria in lung cancer [25], the findings of this study do not support the domination of *Proteobacteria* in tumor tissues. The results of this study showed a significant decrease in *Halomonadaceae*, a family of *Proteobacteria*, and *Halomonas*, a genus of *Proteobacteria*, in tumor tissues of LC patients. Another finding was that the relative abundance of the phylum *Actinobacteria* was significantly decreased in tumor tissues. This finding is consistent with that of Zhuang et al. [32] who reported a decrease of *Actinobacteria* in fecal samples of LC patients. However, this result is contrary to that of Apopa et al. [23] who found an increased level of *Actinobacteria* in LC tissue samples. Apopa et al. also reported an increased abundance of *Proteobacteria*, *Bacteroidetes*, and *Firmicutes*, differing from the results found in our study. The results of this study did not show any significant decrease in *Firmicutes*, as suggested in a study by Greathouse et al. [22]. However, one of its genera, *Lachnoanaerobaculum* was found to be significantly lower in tumor tissues in this meta-analysis. It is interesting to note that in all except one study (Apopa et al.) included in this meta-analysis, a decrease in *Actinobacteria* was observed in tumor tissues of LC patients. This inconsistency may partly be explained by the small sample size of the Apopa et al. study. Decreases in the family *Corynebacteriaceae* and the genus *Corynebacterium* of the phylum *Actinobacteria* were also statistically significant in this study. It has been demonstrated by previous studies that the relative abundance of multiple genera is significantly different in LC patients relative to controls. Hosgood et al. found lower diversity in sputum samples of LC patients which was accompanied by an increase in the relative abundance of *Granulicatella*, *Abiotrophia*, and *Streptococcus* in comparison with healthy controls [20]. Similarly, a lower diversity and an increased abundance of *Streptococcus* was also reported in a study of protected specimen brush (PSB) of malignant parts of the lungs compared to healthy controls [33]. Tsay et al. have reported that *Streptococcus* and *Veillonella* were more abundant in lower airway samples of LC patients compared to patients with benign lung diseases and healthy controls [34]. Yan et al. also found an enriched abundance of the family *Veillonellaceae*, and *Veillonella*, *Capnocytophaga*, and *Selenomonas* genera in the saliva of LC patients [35]. An enriched abundance of *Veillonella* and *Megasphaera* genera was also reported in a study of BAL fluid samples of LC patients compared to patients with benign mass like lesions [19]. Most of available studies have used sputum, PSB, saliva, and BAL fluid specimens to study the microbiome of LC patients rather than a lung biopsy, mainly due to the difficulty of its sampling procedure. But, the high risk of contamination by the upper respiratory tract normal flora associated with the aforementioned sample types should not be ignored, particularly in the case of sputum. In fact, the family *Veillonellaceae* and the genera *Veillonella* and *Streptococcus* are members of the microbial community of the oral cavity and the reports of these genera as differentially abundant taxa between LC patients and controls may be an indication of cross contamination. In this regard, the clinical sample with the lowest risk of contamination by the upper respiratory tract flora is lung biopsy in which samples of lung tissue are isolated from the respiratory tract making it an ideal sample for the lower respiratory system. It is interesting to note that the relative abundance of *Streptococcus* and *Veillonella* was not significantly different between cases and controls in this meta-analysis that may be explained by the fact that all studies included in this meta-analysis used lung biopsy specimens. In a study by Peters et al. lung tumor tissue microbiome has been reported to be less diverse than paired normal tissue [36]. The results of this study showed a significant decrease in the relative abundance of *Halomonas* in tumor tissues. This finding is consistent with that of D’Alessandro-Gabazza et al. who also detected the presence of this genus in the lung tissue of patients with lung cancer as well as idiopathic pulmonary fibrosis [37]. This finding was also reported by Li et al. who showed a difference in the genus *Halomonas* between lung cancer and control groups [38]. All these observations emphasize the need for further investigation of the role of this genus in lung cancer, which may serve as an indicator for prognosis of the disease. Investigating the respiratory microbiome for clinical diagnosis and treatment of respiratory diseases is still in its early stage. In this study, we tried to collectively find a pattern or biomarker based on the composition of the lung microbiome to help differentiate between normal and cancerous conditions. Regarding the poor prognosis of lung cancer and high morbidity and mortality of the disease, development of diagnostic, therapeutic, and prophylactic approaches (especially, probiotics and prebiotics administration) based on microbiome composition is of crucial importance in the field of lung cancer.

To investigate the host-microbiome interactions during disease states, several meta-analyses to date have taken full advantage of NGS technology to discover microbial patterns which are linked to specific diseases, including inflammatory bowel disease (IBD), obesity, COPD, and colorectal cancer. Walters et al. [39] focusing on 16S rRNA gene sequencing studies found a consistent pattern in taxonomic alterations in the gut microbiome of IBD patients. Using supervised learning, they could differentiate IBD from non-IBD individuals. In general, their observations on taxonomic changes were akin to individual studies, including a decrease in *Firmicutes* and *Bacteroides* and an increase in *Proteobacteria* and *Actinobacteria*, though different in several respects. Unlike prior studies, which had reported decreased *Bacteroidetes*, their meta-analysis did not show any statistically significant difference in *Bacteroidetes* phylum between IBD subjects and healthy controls. In addition, considering inconsistencies in the findings of previous microbiome studies regarding the interaction between airway microbiome and host in COPD, Wang et al. [28] analyzed COPD sputum sample microbiome using a total of 15 metagenomic datasets adopting a multi-omic meta-analysis approach. To identify taxonomic alterations in the airway microbiome in COPD versus controls, they limited their meta-analysis by combining the results across two 16S rRNA gene datasets due to the availability of two datasets with the case-control design. Using random-effects meta-analysis, a total of 12 genus-level taxa were identified to be statistically significant between the two groups. Moreover, by training a random forest classifier on COPD datasets, these 12 genera demonstrated to have the potential to distinguish COPD patients from controls. Using an independent multi-omic cohort, they validated their findings indicating that these genera could be considered as the taxonomic signature of airway microbiome in COPD. In fact, these results indicate that some significant associations reported in individual studies may result from insufficient sample sizes and therefore with more statistical power provided with meta-analyses, the consistency and significance of these associations can be assessed more accurately. There are some points concerning performing a meta-analysis on microbiome studies. High heterogeneity of the data generated by high-throughput sequencing of 16S rRNA gene amplicons poses a challenge for inter-study comparisons. To take this matter into account, microbiome data are often standardized to relative abundance data where all microbial taxa range from zero to one. We adopted this standardization approach while combining microbiome data across different studies in the present meta-analysis as it provides greater statistical power in order to identify a core set of microorganisms (core microbiome). A meta-analysis can be conducted adopting different approaches, including aggregate data meta-analyses (AD-MAs) and individual participant data meta-analyses (IPD-MAs). Of the two approaches, combining effect sizes, p-values, and ranks are examples of the former which aggregates summary statistics from individual studies. In the latter case, individual datasets of all included studies are merged into a single dataset. In some respects, a meta-analysis of microbiome data is considered more challenging due to a great deal of variation among microbiome studies. Inherently heterogeneous data place limitations on merging individual datasets into a single dataset. For this reason, although we reprocessed all raw sequence data through a similar pipeline, in this meta-analysis, in an effort to minimize the bias and heterogeneity of data from various sources, we chose to take the first approach rather than directly merging individual datasets as it is more robust to between-study heterogeneity [40]. Specifically, we preferred to combine effect sizes by applying a random-effects model as it has been suggested as a more statistically conservative approach compared to the p-value combination [41, 42]. There are some methodological limitations in this study: (1) it was not possible to determine the relationship between demographic factors (such as age, gender, and smoking history) and taxonomic relative abundance of bacteria due to insufficient metadata. Consequently, we were not able to include them as covariates in the statistical model. To determine how exactly these variables might affect changes in the relative abundance of bacterial taxa, further research with more focus on covariate adjustment is suggested; (2) due to the lack of sufficient data on tumor tissue types, it was not feasible to differentiate lung adenocarcinoma (AC) specific microbiome composition from lung squamous cell carcinoma (SCC) in our analysis; (3) the reported taxa were not significant after adjustment for multiple comparisons mostly due to a considerable number of non-significant associations in this study which can diminish the potential significance of the observed associations. On the one hand, as it has been written about in the literature [43–45], imposing a strict adjustment for multiple comparisons is not always necessary and is less critical in the case of our study since it is not feasible to compare this study’s statistical significance with findings obtained by other studies as there is no large-scale study on the lung microbiome in lung cancer. On the other hand, we acknowledge the need for further investigation to confirm the observed associations in this study; (4) in general, studies identifying the microbial communities at different body sites and determining their relationship with various human diseases belong to an emerging area of research. Initially, the focus of the mentioned studies was mostly on taxonomic profiling of the microbial community. However, research has shown that due to the resilience property of the microbiome (members of the microbiome of a region have some function overlaps and thus different microbiome compositions can have similar overall functions), functional profiling should also be analyzed along with taxonomic profiling. Functional analysis is performed through meta-transcriptomics and metabolomics studies, which is still in its infancy in the case of the respiratory tract microbiome and its association with lung cancer. Therefore, considering the taxonomic composition through 16S rRNA approach is not enough and should be completed by functional analyses in future investigations. Despite the limitations, the present results could have important implications for identifying microbial biomarkers which are linked to the pathogenesis of the disease and facilitate future research. There are still many unanswered questions toward understanding the myriad roles of the lung microbiome and airway host-microbiome interactions with lung cancer. To develop a deeper understanding, further studies, which take the mentioned limitations into account, will be needed. In future investigations, drawing a distinction between different subtypes of Non-Small Cell Lung Cancer (NSCLC) as well as considering the stage of lung cancer could be more informative in characterizing the lung microbial community in LC patients.

## Conclusions

As the first meta-analysis of the lung microbiome in relation to lung cancer, the present research was undertaken to assess how the composition of the lung microbiome differs between lung tumor tissues and normal tissues. The results of this investigation show that some bacteria differ significantly between the two groups. Despite the fact that some of the findings of this study have not previously been described, the results of this research support the idea that the microbiome may be a key factor in cancer development. An important question raised by this study is whether or not these bacterial taxa are specific to lung cancer. To be considered specific indications of lung cancer, the results of this study need to be validated by further research. The insights gained from this study may be of assistance to microbial biomarker discovery and consequently the early diagnosis of lung cancer.

## Methods

### Search Strategy, Inclusion Criteria, and Study Selection

**Fig. 7:**
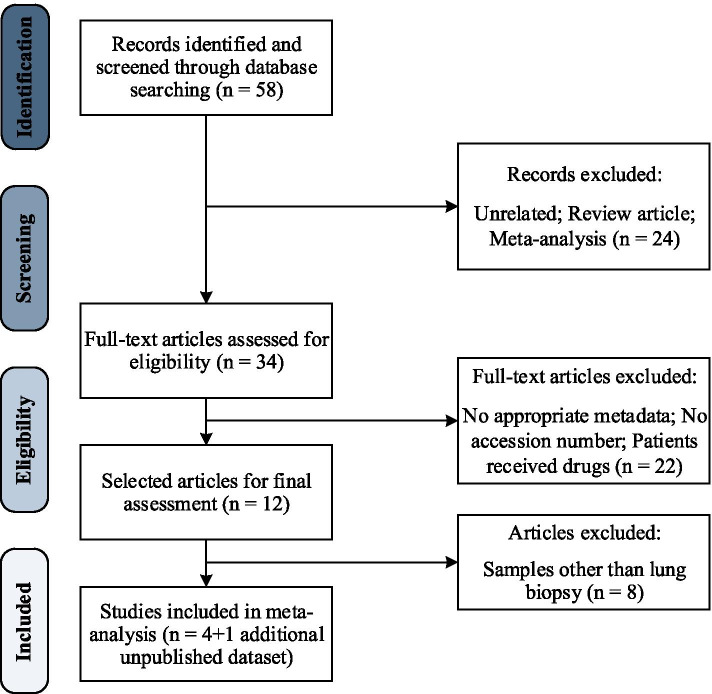
The systematic literature review flow diagram

A systematic literature review was conducted in PubMed, SRA (Sequence Read Archive), and EBI (European Bioinformatics Institute) and was last updated on January 8, 2021. The literature search was based on studies that evaluated the relationship between the microbiome and lung cancer. There was no restriction on the publication date. Published studies were identified using the following keywords: (“lung cancer“[Title/Abstract] OR “lung neoplasm“[Title/Abstract] OR “pulmonary neoplasm“[Title/Abstract] OR “pulmonary cancer“[Title/Abstract]) AND (“microbiome“[Title/Abstract] OR “Metagenom*“[Title/Abstract]). Eligibility criteria required studies to be case-control studies using 16S rRNA gene sequencing for taxonomy quantification of the lung microbial community in LC patients with publicly available raw sequence data. Fifty-eight initially identified articles were reviewed on the basis of titles and abstracts and review articles and meta-analyses were excluded. Full-text of the remaining articles were then assessed and articles with no metadata or accession number along with articles including patients who had received treatment were also excluded. A total of 12 eligible articles meeting our inclusion criteria were selected for further assessment from which 4 studies were chosen for meta-analysis. Eligible studies encompassed a variety of sample types including feces, saliva, BAL fluid, sputum, PSB, and lung biopsy; although due to an insufficient number of studies to perform a meta-analysis, we restricted the sample type to lung biopsy since a total of 5 datasets on lung biopsy specimens were available, one of which (study 4) was unpublished yet obtained from the same population of study 3, as they both had some identical samples. Therefore, due to the small number of available studies on lung biopsy samples of LC patients, we identified the identical samples by subject ID provided in metadata and excluded those samples from study 4 and then considered study 4 as a separate study with its own unique samples. Risk of bias in included studies was also assessed by means of Newcastle-Ottawa Scale (NOS) [46] the results of which are summarized in Table 4. All identified articles were independently assessed by two reviewers. In the case of any discrepancy in study inclusion, a third reviewer was involved and the issue was resolved by discussion. Figure 7 presents an overview of the systematic literature review and Table 5 provides the characteristics of all included studies in this meta-analysis.

**Table 4:** Quality assessment of the included studies in meta-analysis

Study	Risk of bias assessment			
	Selection (0-4)	Comparability (0-2)	Exposure (0-3)	Quality score
Apopa et al.	3	2	3	8
Nejman et al.	3	1	3	7
Yu et al.	3	1	3	7
Kovaleva et al.	3	2	3	8

**Table 5:** Characteristics of the included datasets in meta-analysis

Study	Population	Accession number	16S region	Sequencing platform	CCN	Cases	Controls	Age (y/o)
Study 1 Apopa et al. [23]	AF & EU	PRJNA472758	27 F & 519R	Illumina MiSeq	14	9	5	50–80
Study 2 Nejman et al. [47]	IL	PRJNA624822	*	*	476	245	231	NA
Study 3 Yu et al. [24]	IT 1	PRJNA303190	V3–V4	Illumina MiSeq	257	50	207	40–80
Study 4 -	IT 2	PRJNA327258	NA	Illumina MiSeq	53	27	26	40–80
Study 5 Kovaleva et al. [48]	RU	PRJNA647170	V3–V4	Illumina MiSeq	49	25	24	62$\pm 10$
Total					849	356	493	

CCN: Case-Control Number; Cases: either AC (lung adenocarcinoma) or SCC (lung squamous cell carcinoma); Controls: tumor-adjacent normal tissues; y/o: years old; AF & EU: Africa & Europe; IL: Israel; IT: Italy; RU: Russia; NA: Not Available; * Five regions of the 16S rRNA gene were amplified and sequenced on Illumina HiSeq, MiSeq or NextSeq

### Data Acquisition

Raw sequence data were gathered from NCBI SRA database and corresponding metadata indicating case or control status for each sample were acquired either by search in SRA using the accession number or by personal communication with the authors.

### Data Pre-processing and Taxonomic Profiling

All 16S rRNA marker gene sequencing data were processed through a standardized pipeline in QIIME 2 (version 2020.6) [49]. The first step in this process was to assess the quality of the sequence reads (using FastQC [50]). Paired-end demultiplexed sequences in FASTQ files were quality filtered to identify and remove low-quality reads. Non-biological sequences such as adapters and primers were also separated and trimmed. Running the DADA2 [51] denoising method, paired-end reads were joined. Once QC filtering and the denoising step were completed, denoised data were summarized and an ASV (Amplicon Sequence Variant) table was generated. In the final stage of the process and for the purpose of assigning taxonomy, a Naive Bayes classifier was trained on Greengenes (GG v13.8) ribosomal reference database [52], and taxonomy classification was conducted. This process was carried out independently for all runs of each study. All the parameters used for quality filtering are available in Additional file 1.

### Statistical Analyses

**Fig. 8:**
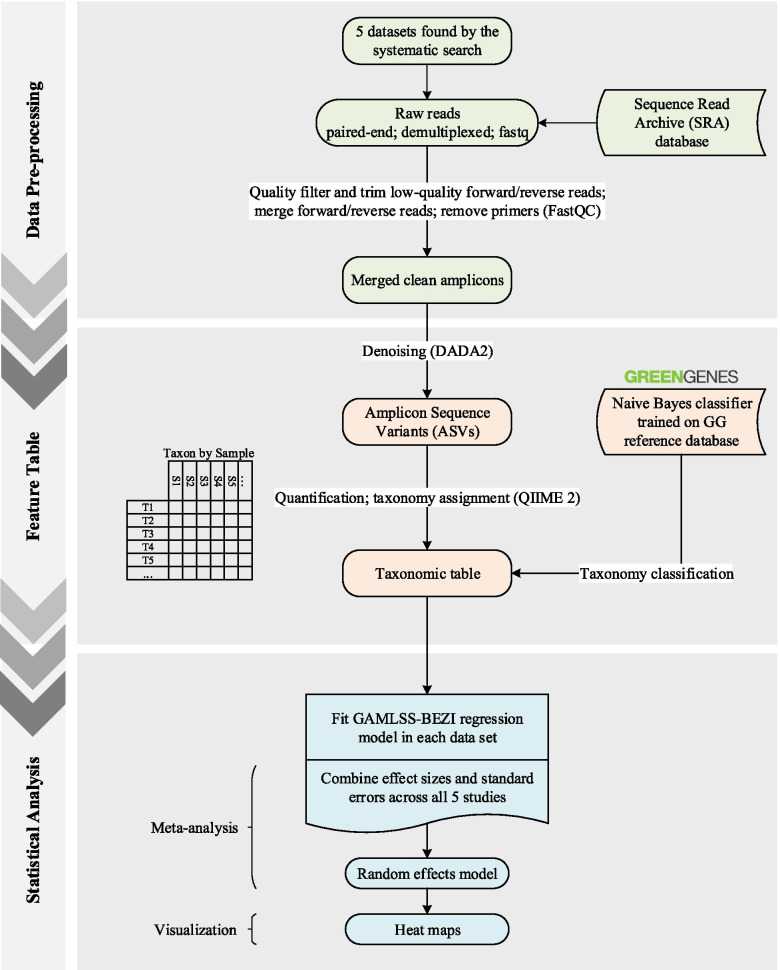
The method workflow for downstream analysis including data pre-processing step, feature table construction, and statistical analyses

The overall workflow is shown in Fig. 8. The analysis was based on the conceptual framework proposed by Ho et al. [53]. Prior to statistical analyses, relative abundance data were obtained by dividing the count value of each taxon by the total counts per sample. Taxonomic relative abundance data were then filtered to remove taxa with the average relative abundance less than $5\times {10}^{-5}$ as well as taxa which were present in fewer than 5 % of samples within each study. The remaining taxa were retained for statistical modeling. All statistical analyses were performed from phylum to genus level within each dataset individually. To compare the relative abundance of the lung bacterial taxa between case and control groups, a GAMLSS-BEZI regression model was fitted in each study; this approach was adopted since it both accurately captures the actual distribution of relative abundance data and specifically addresses zero inflation in microbiome data. The resulting regression coefficient estimate of each taxon from each study (${\beta }_{tk}$) was obtained from GAMLSS-BEZI and considered as effect size and together with its corresponding standard error were retrieved for meta-analysis. If each study contains T taxa, ${\beta }_{tk}$ denotes the effect size for taxon t in study k ($1\le t\le T$ and $1\le k\le K$). To account for inherent heterogeneity among microbiome studies, a random-effects meta-analysis model with inverse variance weighting was then applied to combine calculated effect sizes and their standard errors across all included studies. The following assumption is made by a random-effects model (REM) to combine effect sizes in K studies:

$$\beta_{tk}{\left|\theta_{tk},\;\sigma_{}^{}\right.}_{tk}\sim N\left(\theta_{tk},\sigma_{tk}^2\;\right)$$

$$\theta_{tk}\vert\mu,\tau\sim {N}(\mu,\tau^2)$$

$$\beta_{tk}\left|\mu,\;\tau,\;\sigma_{tk}\sim N\left(\mu,\;\sigma_{tk}^2 +\;\tau^2\right)\right.$$

Then the pooled effect size for ${\beta }_{tk}$ is calculated as follows:

$$\widehat\mu=\frac{\sum_{k=1}^K\;w_{tk}\;\beta_{tk}}{\sum_{k=1}^K\;w_{tk}}$$

Where,

$${w}_{tk}=\frac{1}{{\sigma }_{tk}^{2}+{\widehat{\tau }}^{2}}$$

And ${w}_{tk}$ denotes the weight assigned to study k. ${\tau }^{2}$ is the between-study variance which was estimated based on the DerSimonian and Laird (DL) method.

All taxa available in at least 2 datasets were retained for meta-analysis. Significance levels were set at the 5 % level and all statistical analyses were carried out using R version 4.0.2.

## Supplementary Information

**Additional file 1.** QIIME Quality Filter Parameters. The parameters used in DADA2 denoising step.

## Data Availability

The raw sequence data analyzed during the current study are available in the NCBI SRA repository, https://www.ncbi.nlm.nih.gov/Traces/study/ with the following accession numbers: PRJNA472758, PRJNA624822, PRJNA303190, PRJNA327258, and PRJNA647170. R codes and other materials are available upon request.
